# Clinical Outcomes of Conversion Surgery after FOLFIRINOX in Patients with Unresectable Advanced Pancreatic Cancer: A Retrospective Cohort Study at a Single Center

**DOI:** 10.3390/jcm10132848

**Published:** 2021-06-27

**Authors:** Naoki Mita, Takuji Iwashita, Hironao Ichikawa, Yuhei Iwasa, Shinya Uemura, Katsutoshi Murase, Masahito Shimizu

**Affiliations:** 1 First Department of Internal Medicine, Gifu University Hospital, 1-1 Yanagido, Gifu 501-1194, Japan; mitanao8@yahoo.co.jp (N.M.); ichi.hiro.m.0814@gmail.com (H.I.); festinalenteyu@gmail.com (Y.I.); ueshin550621@gmail.com (S.U.); shimim@gifu-u.ac.jp (M.S.); 2 Department of Digestive Surgery, Gifu University Hospital, Gifu 501-1194, Japan; k_murase@gifu-u.ac.jp

**Keywords:** adjuvant surgery, neoadjuvant therapy, preoperative therapy, pancreatic ductal adenocarcinoma

## Abstract

Pancreatic cancer is one of the most lethal cancers. To improve its prognosis, conversion surgery for initially unresectable advanced pancreatic cancer (UAPC) after chemotherapy has been reported in recent years. Methods: A retrospective analysis of the patients with initially UAPC underwent conversion surgery after the first-line modified FOLFIRINOX (mFX) was conducted at a single tertiary care center between January 2014 and March 2020. Results: Among 79 patients with UAPC who had mFX, 8 patients with a median age of 63 years, including 5 males (3 with locally advanced and 5 metastatic lesions), underwent conversion surgery after a median of 20 cycles of mFX. Conversion surgery was performed in 10.1% of patients (8/79) and surgical resection was successful in all with R0 resection. Postoperative major adverse events were seen in 2 patients, but no perioperative deaths were recognized. Recurrence was confirmed in 3 patients, and these 3 patients died due to cancer recurrence in 17.7, 30.6 and 57.8 months after mFX initiation. 5 patients were still alive without recurrence. The median OS in the patients who underwent conversion surgery was estimated as 65.9 months and was significantly longer than that of the patients without conversion surgery or that in the patients who had a partial response for mFX but did not have conversion surgery. The median follow-up period for the patients who had conversion surgery was 35.2 months. Conclusion: Conversion surgery achieved long-term survival in patients with UAPC who were treated with the first-line mFX, although controversy still remained.

## 1. Introduction

Pancreatic cancer is the fourth leading cause of cancer-related deaths in Japan, with more than 35,000 deaths annually, increasing its proportion in cancer-related deaths over time [1]. Moreover, pancreatic cancer is often diagnosed at an advanced stage with locally advanced and/or metastatic lesions, wherein 10–20% of patients are candidates for surgical resection; despite this, the procedure is the only treatment to achieve complete cure of this disease [2]. It is due to these situations that the prognosis of pancreatic cancer patients is considered poor as compared to other malignant diseases, with an overall five-year survival rate of only 8.5% [1]. 

In cases of unresectable advanced pancreatic cancer, chemotherapy or chemoradiotherapy (CRT) has been the mainstay of treatment, with gemcitabine (GEM) as its standard medication since the late 1990s. Recently, two regimens, FOLFIRINOX (FX; a combination of 5-fluorouracil, oxaliplatin, irinotecan and leucovorin) and GEM plus nab-paclitaxel (GnP) have shown better outcomes than GEM alone in terms of overall survival (OS), progression-free survival (PFS) and response rate (RR). However, the survival benefit is still limited, and the median OS of patients who underwent these new regimens was reported to be within one year [3,4]. To improve pancreatic cancer prognosis, the concept of conversion surgery recently emerged and is surgery for pancreatic cancer, which is initially considered as unresectable advanced one, as a result of significant response to chemotherapy or CRT [5,6,7,8,9,10,11]. However, the efficacy and safety of conversion surgery have not been well studied yet. We then conducted this study to evaluate our experience with conversion surgery following FX regimen for unresectable advanced pancreatic cancer.

## 2. Methods

### 2.1. Study Design and Patient Selection

This was a retrospective study conducted at a single tertiary care center (Gifu University Hospital, Gifu, Japan). Between January 2014 and March 2020, a total of 79 patients with unresectable advanced pancreatic cancer, including locally advanced and metastatic cancer, underwent FX as the first-line chemotherapy at GUH. All patients had pathologically proven pancreatic adenocarcinomas before chemotherapy. In our center, modified FX (mFX; IV oxaliplatin at 85 mg/m^2^ for 2 h, IV leucovorin at 400 mg/m^2^ for 2 h, IV irinotecan at 150 mg/m^2^ for 90 min and IV fluorouracil (5-FU) at 2400 mg/m^2^ over 46 h) has been the first-line regimen for unresectable pancreatic cancer since January 2014, which is followed by gemcitabine plus nab-paclitaxel as the second-line therapy [12,13]. Of the 79 patients, 8 underwent conversion surgery, and their medical records were retrospectively analyzed in this study. The consent for participation of patients in this study was obtained through an opt-out methodology. The study was conducted in accordance with the human and ethical principles of the Helsinki guidelines, and the study protocol was approved by the Institutional Review Board of Gifu University Hospital with the approved number of 2020-148. 

### 2.2. Assessment of Resectability

Initial and final assessment for resectability of pancreatic cancer were evaluated based on findings of dynamic contrast-enhanced computed tomography (CT) either combined with or without dynamic contrast-enhanced magnetic resonance imaging (MRI), endoscopic ultrasound and positron emission tomography (PET) with CT. For the final assessment for resectability in the patients with metastatic lesions, CT, MRI and PET-CT were at least performed for a thorough evaluation. Tumor resectability was determined after discussion by physicians, surgeons and radiologists on a case-by-case basis while referring to the Classification of Pancreatic Cancer by Japan Pancreas Society (4th English Edition) [14]. Pancreatic cancer was essentially deemed unresectable when the following findings were recognized: Metastatic lesion was recognized, or there were major vessel involvements, contacting with the superior mesenteric artery (SMA), celiac artery (CA) or common hepatic artery (CHA) more than 180 degrees, occluding the portal vein (PV) or the superior mesenteric vein (SMV), involving PV or SMV broadly, or involving the aorta. For peritoneal dissemination, the diagnosis was made during initial surgery before mFX and the staging laparoscopy or laparotomy was performed at the time of conversion surgery. 

### 2.3. First-Line Chemotherapy and Assessment of Clinical Outcomes

All patients enrolled in this study underwent mFX as first-line chemotherapy, and none of them underwent CRT. Efficacy of mFX was assessed by comparing CT scans before and after treatment. Conversion surgery indications were also well discussed at a multidisciplinary conference by physicians, surgeons and radiologists on a case-by-case basis.

Different assessments were used for the following outcomes: Response Evaluation Criteria in Solid Tumors (RECIST) was used for radiologic tumor response evaluation; National Cancer Institute Common Terminology Criteria for Adverse Events version 5.0 was used for chemotherapy-related adverse event scoring; the Clavien-Dindo classification was applied used for postoperative complications [15]; the Classification of Pancreatic Cancer (4th English Edition) was used for pathological diagnosis and classification [14], and Evan’s grading system was used for assessing the pathologic effect of preoperative therapy [16].

### 2.4. Study Outcomes and Statistical Analysis

The primary outcome of this study was to evaluate the outcomes of conversion surgery among the cohort underwent mFX for unresectable advanced pancreatic cancer. The secondary outcomes were the rate of conversion surgery and comparison of OS in patients who underwent conversion surgery to the patients who could not.

OS was calculated from the date of mFX initiation to the date of death. The relative dose intensity was calculated as the ratio of the amount of drug that was administered to the amount of standard regimen of mFX until conversion surgery. The OS was estimated using the Kaplan-Meier method and calculated with the corresponding 95% confidence interval (CI). All statistical analyses were performed using JMP 14.0 (SAS Institute, Inc., Cary, NC, USA).

## 3. Results

### 3.1. Patient and Tumor Characteristics

**Figure 1:**
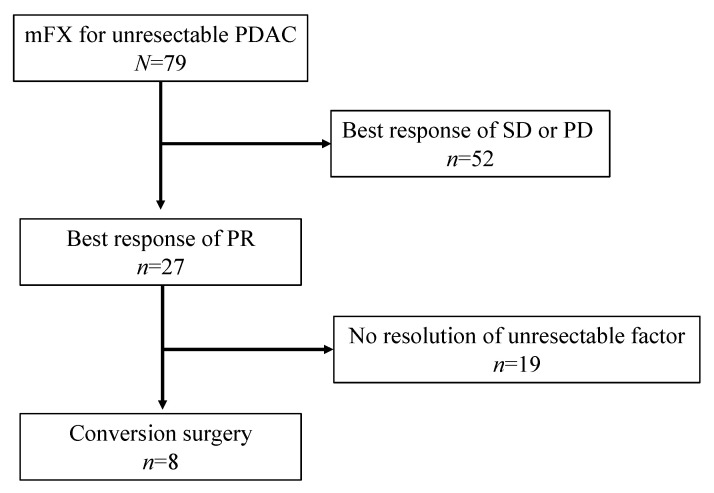
Patient flow.

In this case, 79 patients [median age of 64 year-old (range 38–74); 44 men] underwent mFX for unresectable advanced pancreatic cancer due to local advancement in 20 patients (25.1%) and metastasis in 59 patients (74.9%). Best responses for mFX were partial response in 27 patients (34.2%), stable disease in 32 patients (40.5%) and progressive disease in 20 patients (25.3%). Conversion surgery after the first-line mFX was performed in 8 patients with a resection rate of 10.1%. (Table 1 and Figure 1) The baseline characteristics of the 8 patients including 5 males and 3 females with a median age of 63-year-old (range: 57–72) and are shown in Table 2. The primary tumor site was found to be at the pancreatic head in 4 patients and at the body or tail in the remaining four. The unresectable factors were due to metastatic lesions in 5 patients (3 in the liver, 2 in the peritoneum) and locally advanced lesions in 3 patients (1 in the SMV, 1 in the aorta, and 1 in both the SMA and SMV).

### 3.2. Preoperative Therapy

Detailed information on the preoperative mFX is shown in Table 3. The median duration and cycle were 10.7 months (range: 4.5–23.9) and 20 cycles (range: 6–47), respectively. Grade ≥ 3 chemotherapy-related adverse events were recognized in all patients, including neutropenia in 8 patients and peripheral sensory neuropathy in 1 patient, and the median CA19–9 level was 2921 U/mL (range: 171–10424 U/mL) at peak and 30 U/mL (range: 12–177) after preoperative treatment. The unresectable factors were evaluated using cross-sectional imaging, including contrast-enhanced CT, MRI and PET-CT, to decide indication for conversion surgery, and disappeared in 7 patients. However, in the remaining one (patient #6), major vessel tumor invasion improved, but the perivascular high-density area remained slightly on contrast-enhanced CT.

### 3.3. Surgical Outcomes and Pathological Findings

Regarding the operative procedure for the 8 patients, pancreatoduodenectomy (PD) was performed in 4 patients, distal pancreatectomy (DP) in 3 patients and DP with en bloc celiac axis resection in 1 patient. The median operative time and blood losses were 438 min (range: 220–880) and 648 mL (range: 100–1840), respectively. Gastroduodenal artery pseudoaneurysm rupture and pancreatic fistula occurred in the same patient (Patient #6), and intractable chylous ascites occurred in another (Patient #7), both manifesting as major postoperative complications (Clavien-Dindo class ≥ IIIa). The median hospital stay length was 26.5 days (range: 15–50), and the R0 resection rate was 100%. Regarding the pathological treatment effect based on the Evans grading system [16], Grade IIa was achieved in 2 patients (25%), Grade IIb in 4 patients (50%) and Grade IV in 2 patients (25%) (Table 4).

### 3.4. Adjuvant Chemotherapy and Postoperative Outcomes

**Figure 2:**
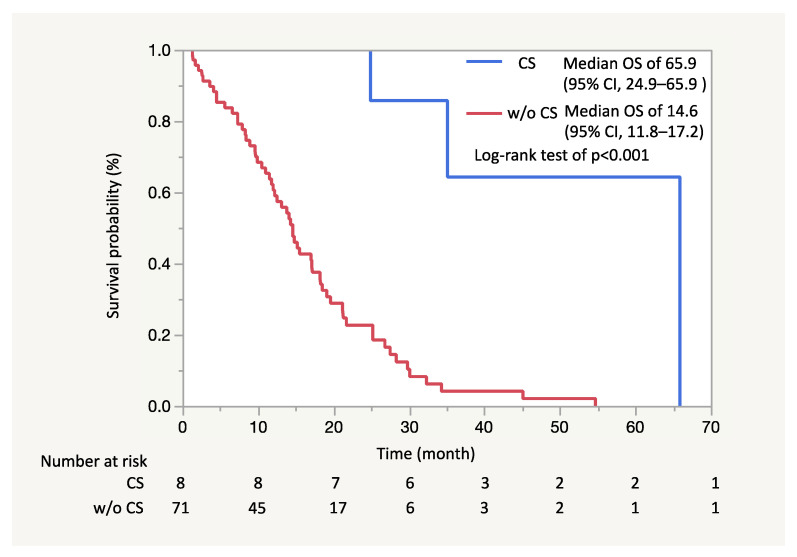
In Kaplan-Meier analysis, the median overall survival (OS) in the patients who underwent conversion surgery was estimated as 65.9 months (95% CI, 24.9–95.9) and was significantly longer than that in all patients without conversion surgery (median OS of 14.6 months; 95% CI, 11.8–17.2) (Log-Rand test of *p* < 0.001).

**Figure 3:**
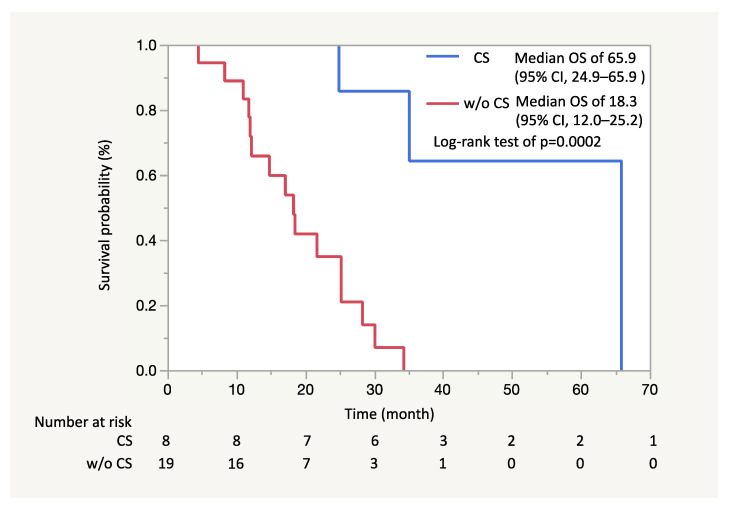
In Kaplan-Meier analysis, the median overall survival (OS) in the patients who underwent conversion surgery was estimated as 65.9 months (95% CI, 24.9–95.9) and was significantly longer than that in the patients who had the best response of partial response but did not have conversion surgery (median OS of 18.3 months; 95% CI, 12.0–25.2) (Log-Rand test of *p* = 0.0002).

Adjuvant chemotherapy was administered in 7 patients (mFX in 4, combination drug of tegafur, gimeracil and oteracil [S1] in 3), and the remaining patient (Patient #6) did not undergo any further treatment. Recurrence was confirmed in 3 (Patient #1, #2 and #7; 37.5%) of the 8 patients. Specifically, Patient #1 had liver metastasis recurrence, Patient #2 recurrence in the remnant pancreas and Patient #7 had lung metastasis recurrence in 8.5, 20.1 and 24.1 months after conversion surgery, respectively. These 3 patients (Patient #1, #2 and #7) were dead in 17.7, 57.8 and 30.6 months after conversion surgery (24.9, 65.9 and 35.1 months from the beginning of mFX), respectively. Overall, 5 patients were still alive without recurrence. (Table 5) The median OS in the patients who underwent conversion surgery was estimated as 65.9 months (95% CI, 24.9–95.9) and was significantly longer than that in all patients without conversion surgery (median OS of 14.6 months; 95% CI, 11.8–17.2) (Figure 2) or that in the patients who had the best response of PR but did not have conversion surgery (median OS of 18.3 months; 95% CI, 12.0–25.2) (Figure 3). The median follow-up period for the patients who had conversion surgery was 35.2 months (range: 24.9–65.9).

## 4. Discussion

The term “conversion surgery” was used to describe surgical resection following chemotherapy for initially unresectable advanced pancreatic cancer in this study. There have been several terminologies to be used for similar treatment strategies, such as “adjuvant surgery,” surgical resection after “neoadjuvant therapy,” or “preoperative therapy” [7]. Natsume et al. further described that “adjuvant surgery” should be used for planned surgeries that are sequentially performed after preoperative neoadjuvant therapy, and “conversion surgery” should be used for unplanned surgeries that are performed as a result of “strategy conversion” due to an unexpected strong anticancer effect with non-surgical therapy [6]. In this study, all 8 patients were considered as non-candidates for surgery before first-line mFX initiation, thus the term “conversion surgery” was considered suitable in this study.

At our institution, mFX was performed as the first-line chemotherapy for unresectable advanced pancreatic cancers, including locally advanced and metastatic cancer, if the patient’s condition allows for it (PS of 0–1 and age of ≤75 years). This treatment strategy enabled evaluation of uniform preoperative therapy using mFX and its treatment effect for conversion surgery. In this study, the conversion surgery rate was 10.1% (8/79) following mFX for unresectable advanced pancreatic cancer. There are several reports on conversion surgery for initially unresectable pancreatic cancer after FX. A retrospective study including 101 locally advanced pancreatic cancer patients by Sadot et al. [17] reported that 31 patients (31%) underwent conversion surgery, and 16 of them (55%) achieved an R0 resection after FX with or without radiation therapy. Another report by Lee et al. [18] also performed a retrospective analysis of 64 patients with locally advanced pancreatic cancer and showed a resection rate of 23% (15/64) and an R0 resection rate of 73% (11/15) after FX with or without radiation therapy. A direct comparison of the conversion surgery rates cannot be performed, since the unresectable factors and procedure indications would be different between studies and institutions. Despite this, the development of more effective chemotherapies, such as FX, for pancreatic cancer has created a new concept of treatment strategy termed “conversion surgery” in a certain number of unresectable pancreatic cancer patients.

**Figure 4:**
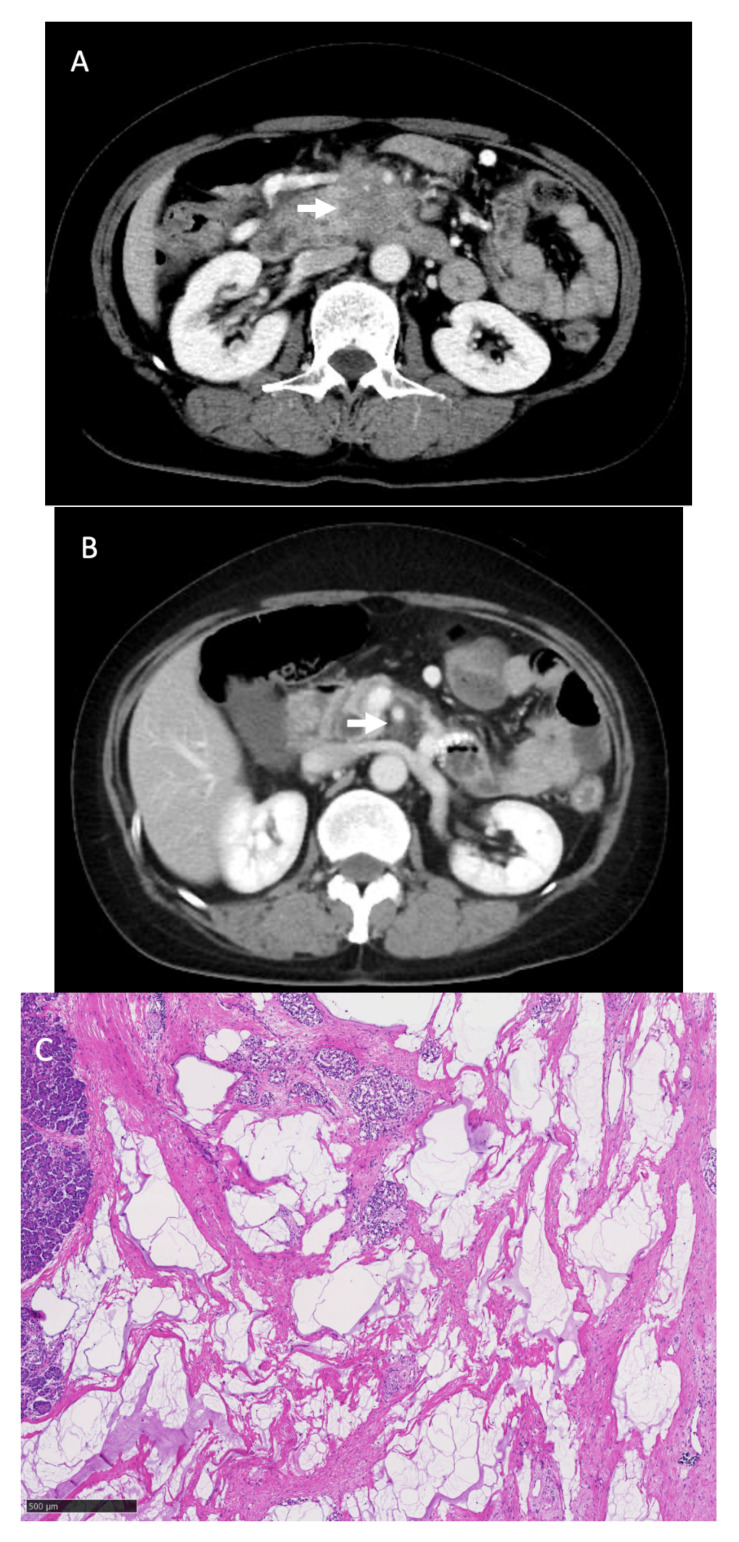
Computed tomography (CT) findings of Patient #6. (**A**): CT findings before chemotherapy showed that the pancreatic head tumor involved the superior mesenteric artery (SMA) (white arrow). (**B**): CT findings before conversion surgery. The high-density area surrounding the SMA (white arrow) remained slightly, although the tumor itself disappeared. (**C**): Pathological findings of the resected tumor showed mainly predominant fibrous tissue with fatty degeneration, mucus pool and remaining Langerhans islands, without any viable tumor cells (hematoxylin and eosin staining). These pathological findings were considered as Evan’s grade of IV.

**Figure 5:**
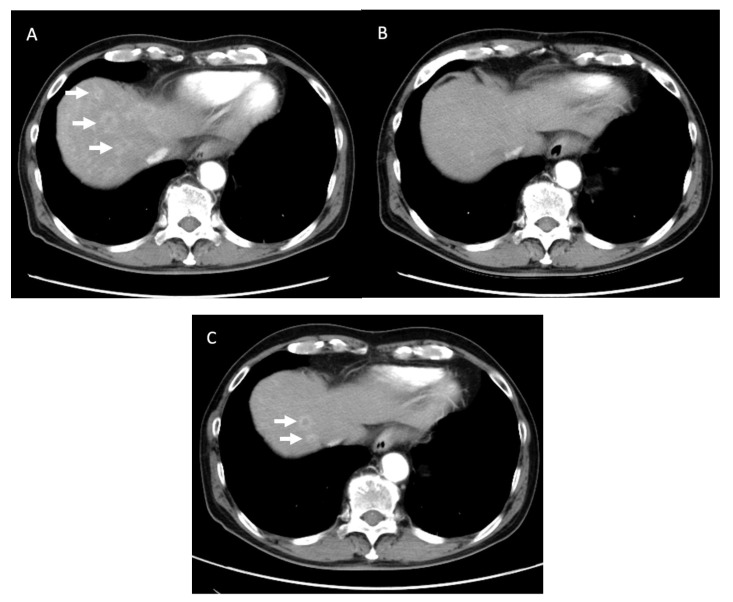
Computed tomography (CT) findings of Patient #1. (**A**): CT showed multiple metastatic lesions (white arrow) in the liver before chemotherapy. (**B**): All liver metastasis disappeared on CT after chemotherapy. (**C**): Cancer recurrence in the liver (white arrow) was confirmed 8.5 months after conversion surgery.

An indication for conversion surgery is patients in whom margin-negative resection could be achieved, although an accurate resectability assessment remains limited based on current imaging modalities. Therefore, its surgical indication seems to be diverse depending on the institution. In some institutions, surgical exploration was offered to evaluate resectability as a more reliable procedure in patients with no progression after chemotherapy [19]. Another indication was that conversion surgery was recommended in patients who were expected to undergo margin-negative resection based on the clinical response [19]. Moreover, the most common indication seems to be decided by clinical response on imaging studies and decreased serum CA19-9 levels [19]. At our institution, the basic indication for conversion surgery is that curative resection could be achieved based on findings of imaging studies, although this is not absolute. In Patient #6, a remarkable response in tumor size and serum CA 19-9 levels was observed, although CT still showed circumferential slightly high-density area around the SMA. However, we decided to perform surgical resection after a multidisciplinary assessment by physicians, surgeons and radiologists, and a discussion with the patient. The final pathological evaluation of the resected specimen only showed a dense desmoplastic change without any viable tumor cells, indicating a complete chemotherapy response (Figure 4). Considering the fibrotic change did not disappear even with CR to mFX in this case, a multidisciplinary assessment is important, because, again, an assessment of resectability based on imaging study is limited. As for an indication of conversion surgery for metastatic one, a more careful assessment might be required. A retrospective cohort study by Tanaka et al. evaluated predictive resectability factors in 101 metastatic pancreatic cancer patients scheduled for surgery after FX, showing a shrinkage rate of the primary tumor of more than 50% (odds ratio [OR]: 13.0, 95% CI: 1.0–162.0, *p* = 0.04) and post-chemotherapy serum CA19-9 level of <150 U/mL (OR: 10.3, 95% CI: 1.4–76.3, *p* = 0.02), which were both significant predictive factors for resectability on multivariate analysis [20]. In this study, among 5 out of 8 patients who underwent conversion surgery for metastatic pancreatic cancer, conversion surgery was decided based on the disappearance of metastatic lesions on imaging studies including CT, MRI and PET-CT. During the study period, 3 patients have not had a recurrence, although 2 patients died because of cancer recurrence (Figure 5). Given all these, at this moment, the indication of conversion surgery for pancreatic cancer should be decided by a comprehensive assessment of the tumor response to chemotherapy based on imaging studies and the serum levels of relevant tumor markers, although further evaluations of its indication are required, especially for metastatic pancreatic cancer.

The optimal preoperative therapy duration also remains controversial. A multicenter retrospective cohort study comparing surgery with non-surgery after a long-term favorable response to non-surgical anti-cancer treatment, which was based on gemcitabine or S1, in initially unresectable pancreatic cancer patients by Satoi et al. performed a subgroup analysis based on the time from initial treatment to surgical resection [21]. On multivariate analysis, their results showed a significantly favorable OS in patients who underwent surgery over 240 days after the initial treatment (hazard ratio: 0.332, 95%CI: 0.150–0.734, *p* = 0.006). Considering the higher response and adverse event rates of FX, we are not sure whether that optimal duration (more than 240 days) could be applicable for FX as well, although a longer duration of chemotherapy may be related to better patient selection. In a retrospective cohort study by Tanaka et al. evaluating predictive resectability factors in 101 metastatic pancreatic cancer patients scheduled for surgery after FX, preoperative FX duration was not a significant predictive factor for longer OS [20]. In our study, 3 patients (Patients #1, #2 and #7) died due to tumor recurrence, although we could not evaluate optimal FX duration for conversion surgery due to the small cohort size. The optimal duration of FX for conversion surgery should also be evaluated in future larger studies.

Regarding the survival benefit of conversion surgery, 5 out of 8 patients were still alive, the median OS in the patients who underwent conversion surgery was estimated as 65.9 months (95% CI, 24.9–95.9) and was significantly longer than that in all patients without conversion surgery. Our previous Phase II study evaluating mFX in 31 unresectable advanced pancreatic cancer patients showed a median OS of 14.9 months (95%CI: 9.9–19.2) [12]. Considering these results, long-term survival was achieved in the patients who underwent conversion surgery; however, the question as to whether conversion surgery contributed to improving the prognosis of patients is unclear. In other words, it is possible that the patients who underwent conversion surgery had a better prognosis than those who received chemotherapy only since these patients should be remarkable responders to preoperative therapy. A multicenter retrospective cohort study comparing surgery with non-surgery after a long-term favorable response to non-surgical anti-cancer treatment in initially unresectable pancreatic cancer patients further found a significantly better overall survival in the conversion surgery group as compared to the control group (median OS: 39.7 months vs. 20.8 months; *p* < 0.0001) [21]. Moreover, Natsume et al. analyzed the survival benefit of conversion surgery by limiting the cohort to responder patients for the first-line therapy, reporting that the survival rate was better in patients who underwent conversion surgery than in those who did not (median OS: not reached vs. 562 days, *p* < 0.001) [6]. In our study as well, the median OS in the patients who underwent conversion surgery was significantly longer than that in the patients who had the best response of PR but did not have conversion surgery (65.9 vs. 18.3 months). These results are supporting the survival benefits of conversion surgery in patients with unresectable advanced pancreatic cancer, although the benefit cannot be completely confirmed. Further studies are needed to evaluate the survival benefit of conversion surgery as well.

The major limitation of our study was that the study included a small number of patients who underwent conversion surgery at a single center. Due to small cohort size, the outcomes could not be separately evaluated between locally advanced and metastatic advanced pancreatic cancer, although these two conditions might be different. An insufficient follow-up period was also another limitation. However, the strength of our study was that all preoperative treatments were performed with a unified regimen (mFX) by a single dedicated team of specialized pancreaticobiliary physicians.

In conclusion, long-term survival was achieved in initially unresectable advanced pancreatic cancer patients, including those with locally advanced and metastatic cancer, treated with the first-line mFX followed by conversion surgery. Despite these findings, optimal treatment regimen, optimal preoperative treatment duration and optimal surgery indications remain controversial. Therefore, further large-scale studies are required to evaluate these points.

## Figures and Tables

**Table 1:** Basic characteristic of all 79 patients.

Age, Years	Median (Range)		64 (38–74)
Sex	n (%)	men	44 (55.7)
Metastasis	n (%)	yes	59 (74.7)
Best response based on imaging studies	n (%)	CR	0
		PR	27 (34.2)
		SD	32 (40.5)
		PD	20 (25.3)
Conversion surgery	n (%)		8 (10.1)

CR, complete response; PR, partial response; SD, stable disease; PD progressive disease.

**Table 2:** Basic characteristic of patients underwent conversion surgery.

Patient No.	Age (years)	Gender	Site of Primary Tumor	Unresectable Factor
1	63	Male	Tail	M (HEP)
2	72	Male	Head	LA (SMV)
3	59	Male	Body	M (PER)
4	63	Female	Body	M (HEP)
5	58	Male	Head	LA (Ao)
6	57	Female	Head	LA (SMA, SMV)
7	63	Male	Body	M (PER)
8	63	Female	Head	M (HEP)

M, metastatic lesion (HEP, liver; PER, Peritoneal); LA, locally advanced lesion; (SMV, superior mesenteric vein; Ao, aorta; SMA, superior mesenteric artery).

**Table 3:** Details and effects of preoperative chemotherapy.

Patient No.	Duration (Months) (Cycles)	Treatment Effect (RECIST)	Adverse Events (≥Grade 3)	CA19–9(U/mL) Peak/after Chemotherapy	Relative Dose Intensity (%)				Findings of Unresectable Factor	
					L-OHP	CPT-11	5-FU	LV		
1	7.1 (10)	PR	Neutropenia G4	6529 / 32	42.9	56.2	68.1	68.1	M (HEP/Multi)	disappeared
2	8.0 (13)	PR	Peripheral sensory neuropathy G3	1462 / 55	59.6	74.5	85.0	85.0	LA (SMV)	disappeared
3	23.9 (47)	PR	Neutropenia G3	250 / 13	65.1	79.8	86.8	87.7	M (PER/Multi)	disappeared
4	23.3 (31)	PR	Neutropenia G4	4379 / 81	42.8	56.4	69.9	68	M (HEP/Multi)	disappeared
5	6.8 (11)	PR	Neutropenia G4	171 / 12	71.8	81.4	83.4	83.0	LA (Ao)	disappeared
6	13.3 (27)	PR	Neutropenia G4	5431 / 20	60.9	89.7	92.7	92.2	LA (SMA, SMV)	improved
7	4.5 (6)	PR	Neutropenia G3	370 / 177	50.2	53.3	65	66	M (PER/Multi)	disappeared
8	24.0 (36)	PR	Neutropenia G4	10424 / 27	41.7	56.5	65.7	66.4	M (HEP/Multi)	disappeared

RECIST, response evaluation criteria in solid tumors; L-OHP, oxaliplatin; CPT-11, irinotecan; 5-FU, fluorouracil; LV, leucovorin; PR, partial response; G, grade; M, metastatic lesion (HEP, liver; PER, Peritoneal); Multi, multiple metastasis sites; LA, locally advanced lesion (SMV, superior mesenteric vein; Ao, aorta; SMA, superior mesenteric artery).

**Table 4:** Surgical outcomes and pathological findings.

Patient No.	Operative Procedure	Operative Time (min)	Blood Loss (ml)	Major Complications	Hospital Stay (Days)	pTNM	Residual Tumor	Tumor Viability (Evans)
1	DP	220	100	-	20	pT2N0M0, stageIB	R0	IIb
2	PD	520	710	-	50	pT2N1M0, stageIIB	R0	IIb
3	DP	445	1840	-	22	pT3N0M0, stageIIA	R0	IIa
4	DP	350	290	-	19	pT3N0M0, stageIIA	R0	IIb
5	PD	500	685	-	31	pT3N0M0, stageIIA	R0	IIb
6	PD	880	1790	GDA pseudoaneurysm rupture Pancreatic fistula	47	pCR	R0	IV
7	DP-CAR	360	330	-	15	pT3N1M0, stageIIB	R0	IIb
8	PD	430	610	Chylous ascites	41	pCR	R0	IV

DP, distal pancreatectomy; PD, pancreatoduodenectomy; DP-CAR, distal pancreatectomy with en bloc celiac axis resection; GDA, gastroduodenal artery; pCR, pathologically complete response.

**Table 5:** Adjuvant Chemotherapy and postoperative outcomes.

Patient No.	Adjuvant Chemotherapy	Recurrence Site/Time to Recurrence from CS (Month)	Final Outcome	OS (Months) from Initial Treatment [from Surgery]
1	mFX	Liver/8.5	Death	24.9 [17.7]
2	S1	Remnant pancreas/20.1	Death	65.9 [57.8]
3	mFX	No	Alive	40.9 [16.7]
4	mFX	No	Alive	35.2 [12.0]
5	mFX	No	Alive	26.1 [19.3]
6	No	No	Alive	27.3 [13.9]
7	S1	Lung/24.1	Death	35.1 [30.6]
8	S1	No	Alive	26.7 [12.6]

OS, overall survival; mFX, modified FOLFIRINOX; S1, the combination drug of tegafur, gimeracil and oteracil; CS, conversion surgery; D/A, dead/alive.

## Abbreviations

mFX, modified FOLFIRINOX; CRT, chemoradiotherapy; GEM, gemcitabine; GnP, gemcitabine plus nab-paclitaxel; OS, overall survival; PFS, progression free survival; RR, response rate; SMA, superior mesenteric artery; CA, celiac artery; CHA, common hepatic artery; SMV, superior mesenteric vein; CT, computed tomography; RECIST, response evaluation criteria in solid tumors; MRI, magnetic resonance imaging; PET-CT, positron emission tomography CT; PD, pancreatoduodenectomy; DP, distal pancreatectomy; pCR, pathological complete response.
